# Targeting Src attenuates peritoneal fibrosis and inhibits the epithelial to mesenchymal transition

**DOI:** 10.18632/oncotarget.20040

**Published:** 2017-08-08

**Authors:** Jun Wang, Li Wang, Liuqing Xu, Yingfeng Shi, Feng Liu, Hualin Qi, Na Liu, Shougang Zhuang

**Affiliations:** ^1^ Department of Nephrology, Shanghai East Hospital, Tongji University School of Medicine, Shanghai, China; ^2^ Department of Medicine, Alpert Medical School and Rhode Island Hospital, Brown University, Providence, RI, USA

**Keywords:** Src, peritoneal fibrosis, epithelial to mesenchymal transition, myofibroblasts, epidermal growth factor receptor

## Abstract

Src has been reported to mediate tissue fibrosis in several organs, but its role in peritoneal fibrosis remains unknown. In this study, we evaluated the therapeutic effect of KX2-391, a highly selective inhibitor of Src, on the development of peritoneal fibrosis in a rat model. Daily intraperitoneal injections of chlorhexidine gluconate induced peritoneal fibrosis, as indicated by thickening of the submesothelial area with an accumulation of collagen fibrils and activation of myofibroblasts. This was accompanied by time-dependent phosphorylation of Src at tyrosine 416. Administration of KX2-391 attenuated peritoneal fibrosis and abrogated increased phosphorylation of Src and multiple signaling molecules associated with tissue fibrosis, including epidermal growth factor receptor, Akt, Signal transducer and activator of transcription 3 and nuclear factor-κB in the injured peritoneum. KX2-391 also inhibited the production of proinflammatory cytokines and the infiltration of macrophages into the injured peritoneum. In cultured human peritoneal mesothelial cells, inhibition of Src by KX2-391 or siRNA resulted in decreased expression of α-smooth muscle actin (α-SMA), fibronectin and collagen I, the hallmarks of epithelial to mesenchymal transition. These results suggest that Src is a critical mediator of peritoneal fibrosis and the epithelial to mesenchymal transition. Thus, Src could be a potential therapeutic target in the treatment of peritoneal fibrosis.

## INTRODUCTION

Peritoneal dialysis (PD) has been widely used in patients with end-stage renal disease. During PD, the peritoneal membrane (PM) is continually exposed to hyperosmotic, hyperglycemic and acidic dialysis solutions as well as mechanical stress. Over time, these stimuli may cause injury to the PM, resulting in peritoneal fibrosis, which is characterized by differentiation of fibroblasts to myofibroblasts, overproduction of extracellular matrix (ECM) components and neovascularization [1, 2]. The thickened submesothelial fibrotic layer due to ECM deposition in the peritoneum results in a decline in peritoneal membrane function [3] and eventual ultrafiltration failure [4]. To date, there is no available approach to prevent or halt the development and progression of peritoneal fibrosis, it thus is important to identify the factors and mechanisms that contribute to this process and to develop new antifibrotic therapies.

During the development of peritoneal fibrosis, many cytokines and growth factors are produced and multiple profibrotic signaling pathways are activated. Specifically, TGF-*β*1 is considered the main mediator of peritoneal fibrosis. TGF-β1 binding to its receptor and induces phosphorylation of Smad3, a key signaling molecule, which promotes expression of genes associated with fibrosis such as collagen I [5, 6]. Activation of the TGF-β1/Smad3 pathway also triggers the epithelial to mesenchymal transition (EMT) of peritoneal mesothelium cells, activation of fibroblasts and angiogenesis in the rodent peritoneum [5]. In addition, increased expression and activation of multiple receptor tyrosine kinases (RTK), including epidermal growth factor receptor (EGFR), platelet-derived growth factor receptor (PDGFR), fibroblast growth factor receptor (FGFR), and vascular endothelial growth factor receptor (VEGFR), have been identified in the peritoneum undergoing peritoneal fibrosis [7]. Although the role and mechanisms of these RTKs in peritoneal fibrosis remains poorly understood, our recent studies have shown that EGFR is critically involved in the progression of peritoneal fibrosis [8].

Inflammation and angiogenesis are also important mechanisms in the development of peritoneal fibrosis [5]. In PD patients, many factors such as bioincompatible PD solution, peritonitis and uremia can induce inflammation through macrophage activation [5]. Activated macrophages produce numerous proinflammatory cytokines, including interleukin-1β (IL-1β), IL-6, IL-8, tumor necrosis factor (TNF)-α and monocyte chemoattractant protein (MCP)-1 [9, 10]. Moreover, peritoneal vascular endothelial cells and vascular smooth muscle cells can produce vascular endothelial growth factor (VEGF) [11] that leads to increased endothelial permeability and angiogenesis [12].

Activation of growth factor receptors initiates phosphorylation of multiple intracellular signaling molecules including Src, which is a non-receptor tyrosine kinase that regulates a multitude of biologic events, including cell survival, proliferation and migration [13]. It can be activated by autophosphorylation at Tyr416 in response to many stimuli including growth factors, cytokines and vascular active substances [13]. Activated Src triggers subsequent signal transduction and gene expression through activation of several intracellular signaling pathways such as signal transducer and activator of transcription (STAT3) and AKT [14–16]. Interestingly, Src has been shown to induce EGFR transactivation and production of TGF-β1 in response to stimulation with angiotensin II [17].

Src was originally identified as the first proto-oncogene [16]. Growing evidence has revealed that Src is also involved in the pathogenesis of fibrotic disorders in several organs such as dermal fibrosis [18], bleomycin-induced lung fibrosis [19], hepatic fibrosis [20] , and renal fibrosis [21–23]. Recently, nintedanib, an inhibitor that targets multiple RTKs as well as Src kinases has been approved to treat patients with idiopathic pulmonary fibrosis [24], suggesting that Src could be a therapeutic target in fibrotic diseases. However, it remains unknown whether Src is activated in the peritoneum following PD and involved in the development of peritoneal fibrosis.

In this study, we investigated the role of Src in the epithelial to mesenchymal transition of cultured human peritoneal mesothelial cells as well as in a rat model of peritoneal fibrosis induced by chlorhexidine gluconate (CG).

## RESULTS

### Src is activated during the development of peritoneal fibrosis induced by CG injury

**Figure 1:**
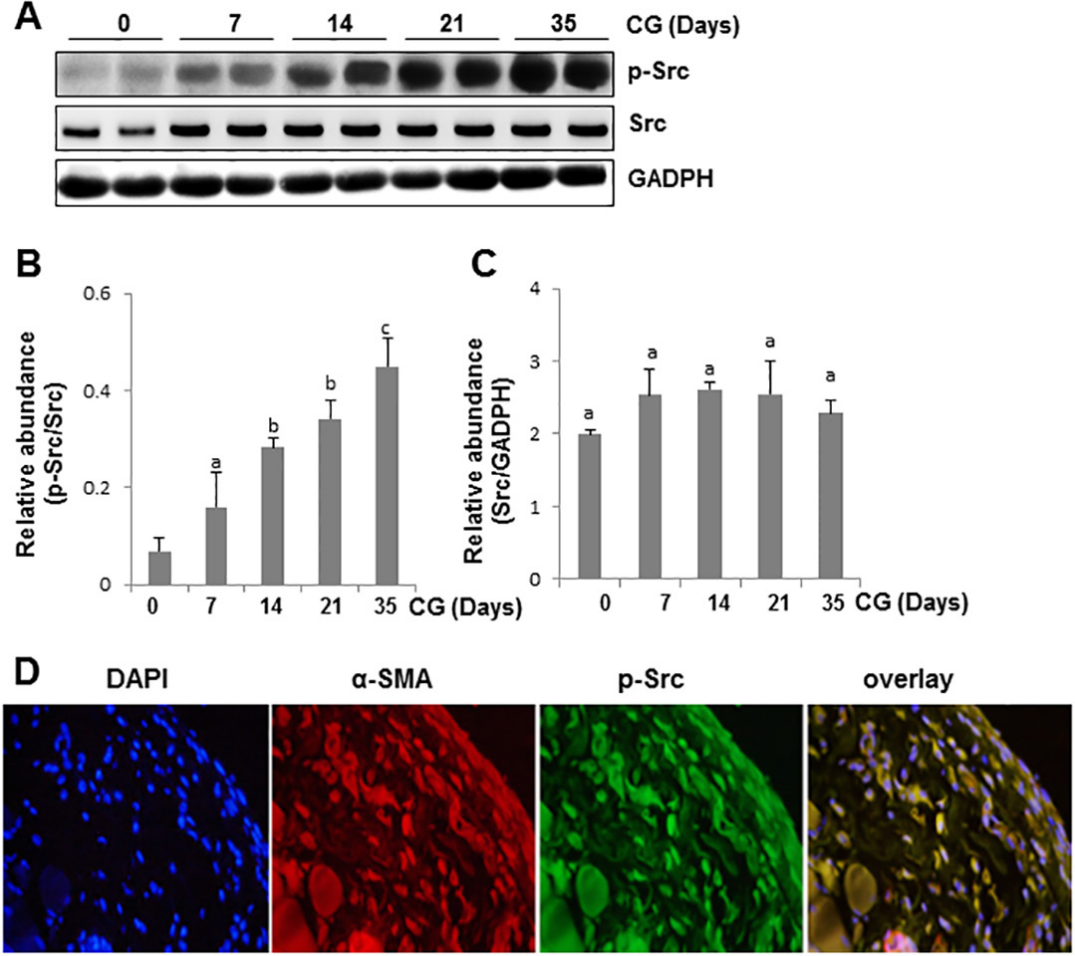
Time course of CG-induced expression of phospho-Src and total Src in the peritoneum (**A**) Peritoneal membrane lysates were subject to immunoblot analysis with specific antibodies against p-Src, Src or GADPH. (**B**) Expression levels of p-Src were quantified by densitometry and normalized with Src. (**C**) Expression levels of Src were quantified by densitometry and normalized with GADPH. Data are represented as the mean ± SEM (*n* = 6). Bars with different letters (a–c) are significantly different from one another (*P* < 0.05). (**D**) Photomicrographs illustrate co-staining of α-SMA and p-Src in the peritoneum collected 21 days after CG injection. DAPI, 4′,6-diamidino-2-phenylindole.

Src can be activated by its phosphorylation at Tyr416 in response to numerous growth factor, cytokines and other stimuli [13]. To determine whether Src would be activated in peritoneal fibrosis, we examined expression of phospho-Src (p-Src) at Tyr416 and total Src in the peritoneum over time in a rat model of peritoneal fibrosis induced by chlorhexidine gluconate (CG). As shown in Figure 1, injury to peritoneum with CG injection induced Src tyrosine phosphorylation at Tyr416, which occurred as early as 7 days, gradually increased, and peaked at 35 days (Figure 1A and 1B), whereas the total Src level was not altered during the course of peritoneal fibrosis (Figure 1A and 1C). Immunostaining showed that p-Src is expressed most abundantly in the α-SMA positive cells (Figure 1D). These results illustrated that CG-induced peritoneal fibrosis was accompanied by persistent activation of Src and that p-Src is preferentially co-localized within cells expressing α-SMA in the peritoneum.

### Src inhibition attenuates development of peritoneal fibrosis in the peritoneum after CG injury

**Figure 2:**
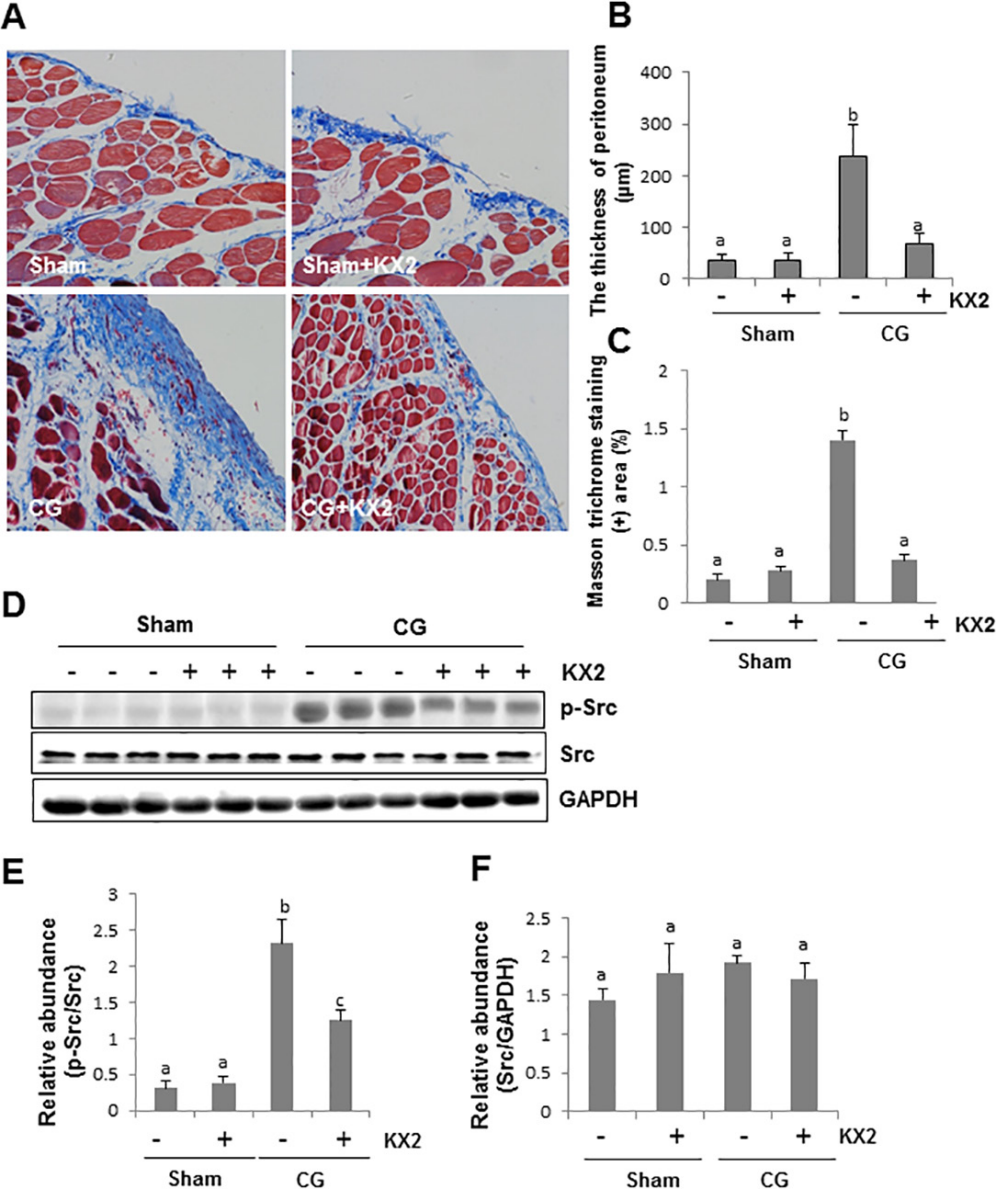
KX2-391 attenuates development of CG-induced peritoneal fibrosis Peritoneal membrane was collected at 21 days after CG injury with or without administration of KX2-391 (KX2) (**A**–**F**). (A) Photomicrographs illustrate Masson trichrome staining of the peritoneum. (B) The graph shows the thickness of the compact zone measured from 10 random fields (200 ×) of six rat peritoneal samples. (C) The graph shows the score of the Masson-positive submesothelial area (blue) from 10 random fields (200 ×) of six rat peritoneal samples. (D) The peritoneal tissue lysates were subjected to immunoblot analysis with specific antibodies against p-Src, Src, or GAPDH. (E) Expression levels of p-Src were quantified by densitometry and normalized with Src. (F) Expression levels of Src were quantified by densitometry and normalized with GAPDH. Data are means ± SEM (*n* = 6). Bars with different letters (a–c) are significantly different from one another (*P* < 0.05).

To assess whether Src activation contributes to development of peritoneal fibrosis, 5 mg/kg of KX2-391, a highly selective Src inhibitor that is orally bioavailable and under clinical trials for tumors [25, 26], was administered immediately after CG injection at day 1 and then given daily for 21 days. As shown in Figure 2A, 2B, the thickness of the submesothelial zone in CG-injured rats treated with KX2-391 was significantly less than that in rats subjected to CG alone. KX2-391 treatment also reduced the area of collagen fibrils in the submesothelial compact zone (Figure 2C). Treatment with KX2-391 significantly reduced CG-induced Src phosphorylation in the peritoneum (Figure 2D and 2E). However, expression levels of total Src were not affected by this agent (Figure 2D and 2F). These data indicated that KX2-391 is a potent agent for inactivation of Src and inhibition of peritoneal fibrosis.

### Src inhibition reduces activation of fibroblasts and deposition of ECM in the peritoneum after CG injury

**Figure 3:**
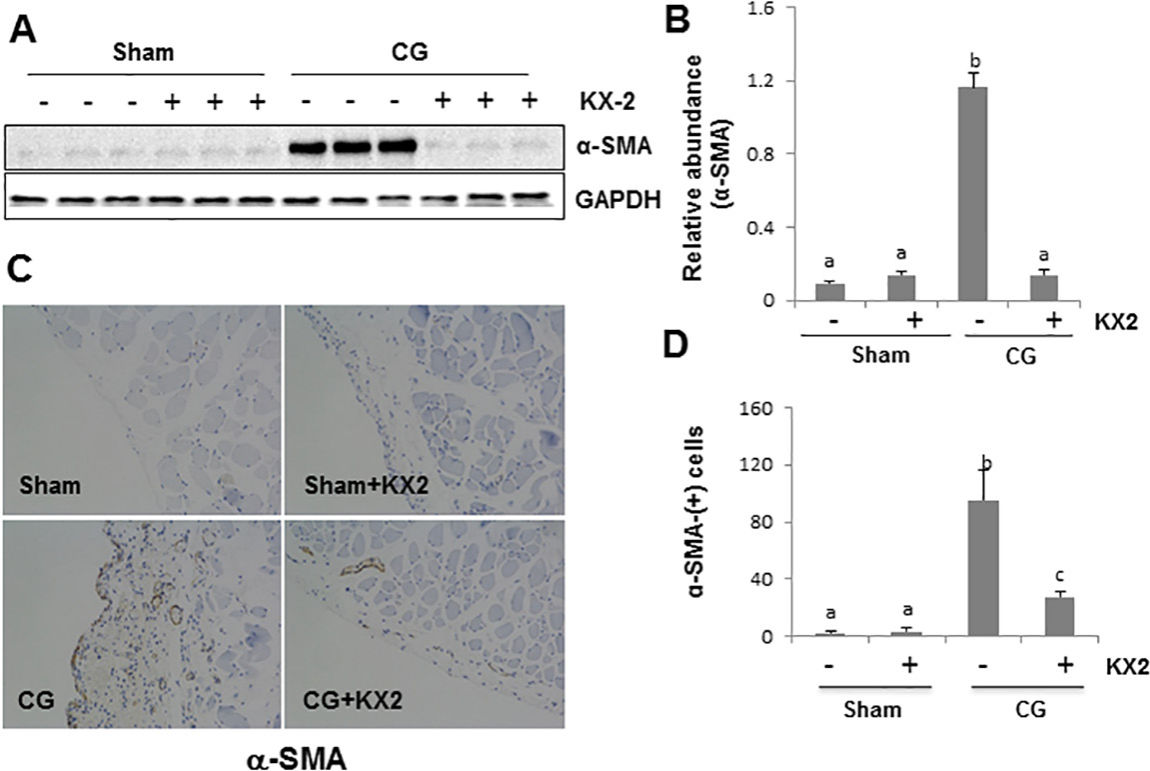
KX2-391 inhibits activation of fibroblasts in the injured peritoneum Peritoneal membrane was collected at 21 days after CG injury with or without administration of KX2-391(KX2) (**A**–**D**). (A) The peritoneal tissue lysates were subjected to immunoblot analysis with specific antibodies against α-SMA or GAPDH. (B) Expression levels of α-SMA were quantified by densitometry and normalized with GAPDH. (C) Photomicrographs illustrate immunohistochemical staining of α-SMA in the submesothelial compact zone. (D) The number of α-SMA positive cells was calculated from ten random fields of six rat peritoneal samples. Data are means ± SEM. (*n* = 6). Bars with different letters (a–c) are significantly different from one another (*P* < 0.05).

To confirm the above observations, we examined the ability of KX2-391 in suppressing fibroblast activation in this model. As shown in Figure 3A and 3B, KX2-391 treatment significantly inhibited expression of α-SMA, a hallmark of activated fibroblasts (myofibroblasts) in the peritoneum. Immunohistochemistry staining also showed that KX2-391 treatment reduced the number of α-SMA positive cells in the submesothelial compact zone (Figure 3C and 3D).

**Figure 4:**
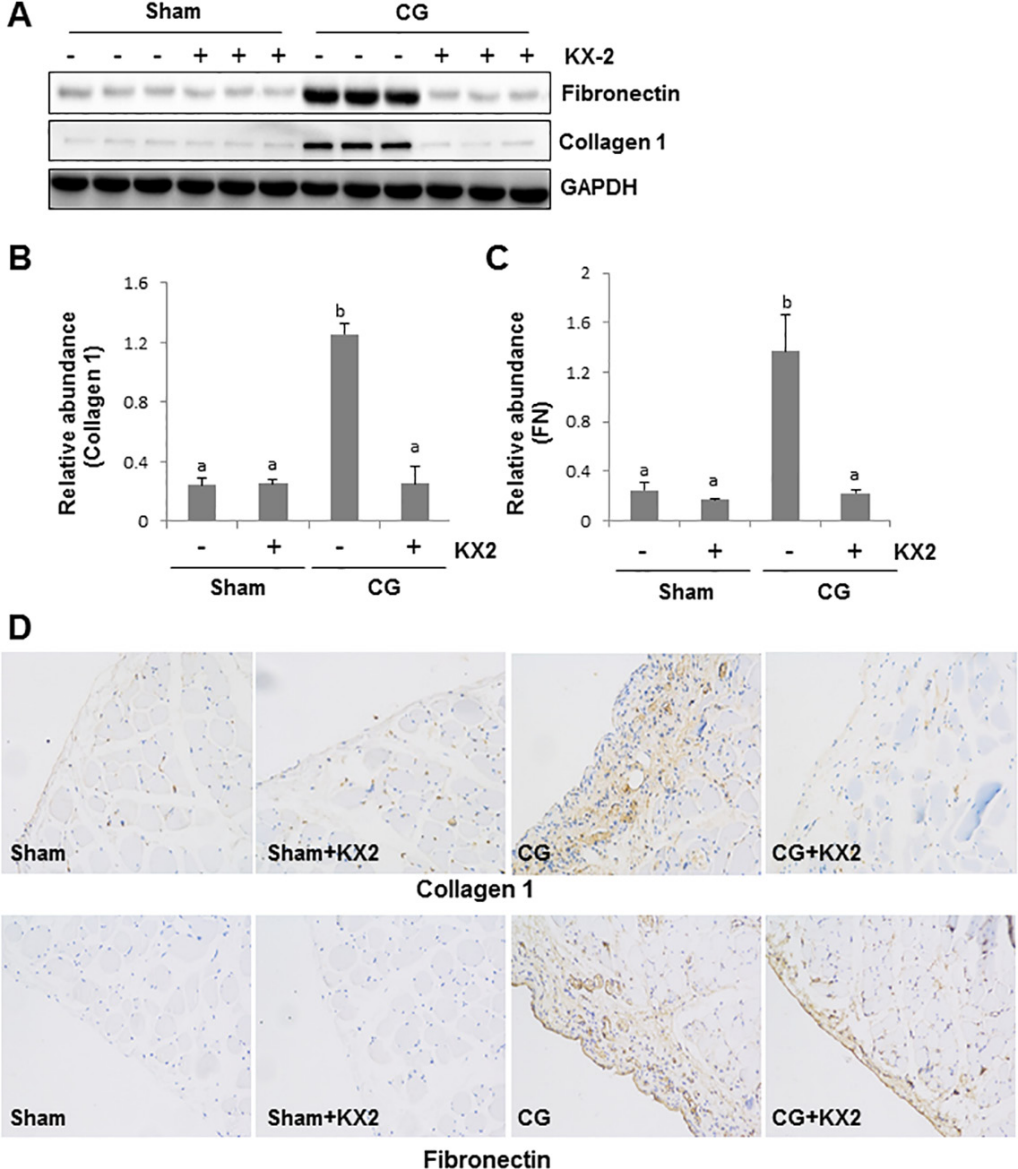
KX2-391 inhibits deposition of ECM in the injured peritoneum Peritoneal membrane was collected at 21 days after CG injury with or without administration of KX2-391(KX2) (**A**–**D**). (A) The peritoneal tissue lysates were subjected to immunoblot analysis with specific antibodies against collagen 1, fibronectin or GAPDH. Expression levels of collagen 1(B) and fibronectin (C) were quantified by densitometry and normalized with GAPDH, respectively. Data are means ± SEM. (*n* = 6). Bars with different letters (a–b) are significantly different from one another (*P* < 0.05). (D) Photomicrographs illustrate immunohistochemical staining of collagen 1 and fibronectin (yellow) in the submesothelial compact zone.

As myofibroblasts are a major cell type to produce ECM proteins, we also assessed whether KX2-391 would be effective in suppressing the deposition of ECM in the peritoneum. As shown in Figure 4A–4C, treatment with KX2-391 inhibited CG-induced expression of collagen 1 and fibronectin to the basal level as evidenced by immunoblot analysis. Immunohistochemistry staining further confirmed that KX2-391 treatment reduced expression levels of collagen 1 and fibronectin in the submesothelial compact zone (Figure 4D). This data, together with the results from Figures 2 and 3, indicate that Src activation is critically involved in the development of peritoneal fibrosis induced by CG.

### Src activates TGF-β1 signaling pathway in the peritoneum after CG injury

**Figure 5:**
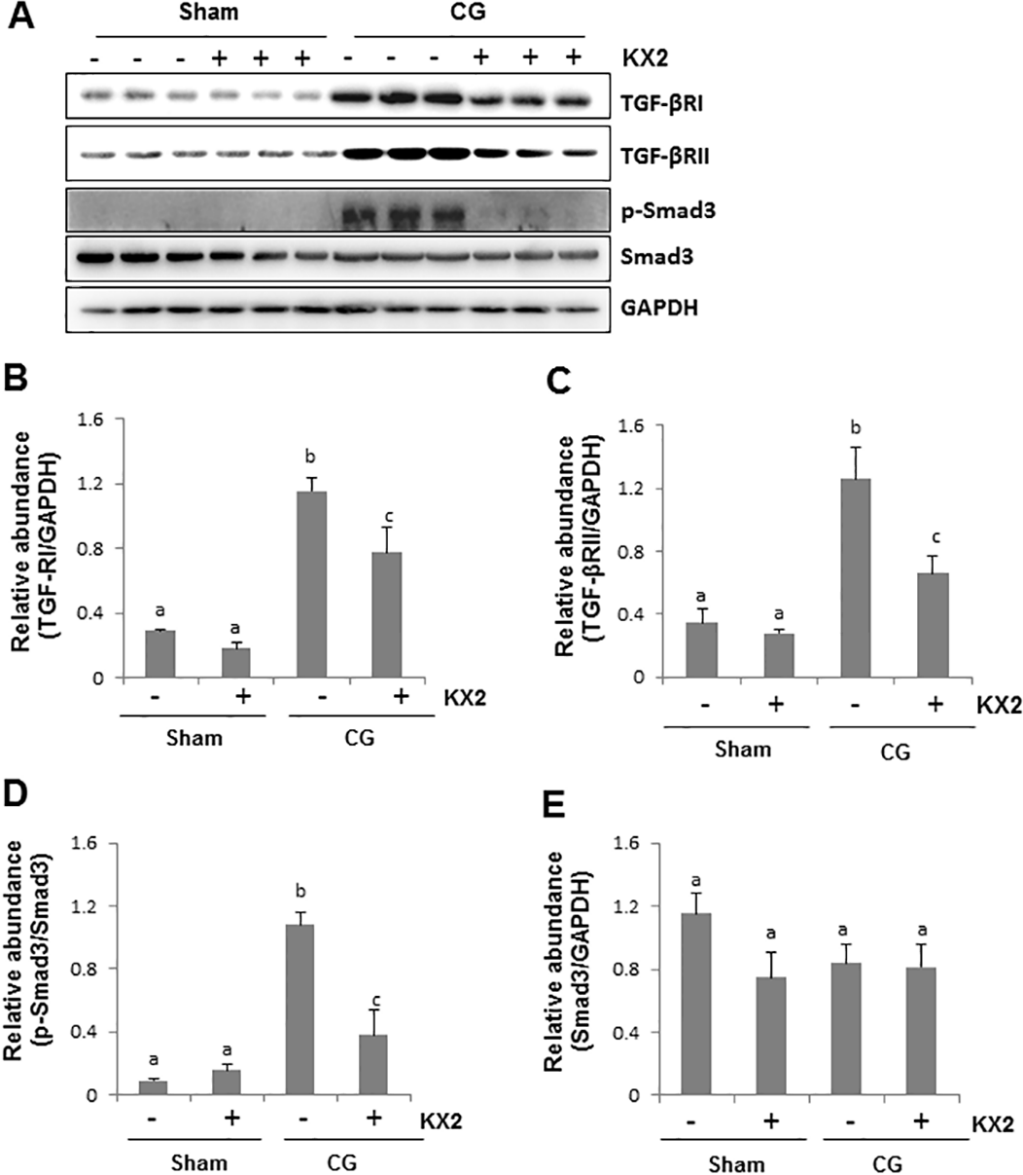
KX2-391 inhibits expression of TGF-β receptor I and II as well as activation of Smad3 in the injured peritoneum Peritoneal membrane was collected at 21 days after CG injury with or without administration of KX2-391(KX2) (5 mg/kg, daily). (**A**) Peritoneum tissue lysates were subjected to immunoblot analysis with specific antibodies against TGF-β receptor I (TGF-βRI), TGF-β receptor II (TGF-βRII), p-Smad3 and Smad3. Expression levels of TGF-βRI (**B**), TGF-βRII (**C**), and Smad3 (**E**) were quantified by densitometry and normalized with GAPDH, respectively. (**D**) Expression levels of p-Smad3 were quantified by densitometry and normalized with Smad3. Bars with different letters (a–c) are significantly different from one another (*P* < 0.05).

Activation of TGF-β1 signaling is key to the development and progression of peritoneal fibrosis [26]. To explore the mechanism by which Src activation contributes to peritoneal fibrosis, we examined the effect of Src inhibition on the expression of TGF-β receptor I and II as well as the phosphorylation (activation) of Smad3 in the peritoneum after CG injury. As shown in Figure 5A–5C, the expression level of both TGF-β receptor I and II markedly increased after CG injection, whereas inhibition of Src by KX2-391 reduced their expression. CG treatment also increased the expression of phospho-Smad3 (p-Smad3), and KX2-391 treatment suppressed its expression (Figure 5A, 5D). Notably, expression of total Smad3 was not affected by KX2-391 (Figure 5A, 5D, 5E). These results suggest that the antifibrotic effect of KX2-391 is associated with the suppression of the TGF-β/Smad signaling pathway in CG-induced peritoneal fibrosis.

### Src contributed to the activation of EGFR in the peritoneum after CG injury

**Figure 6:**
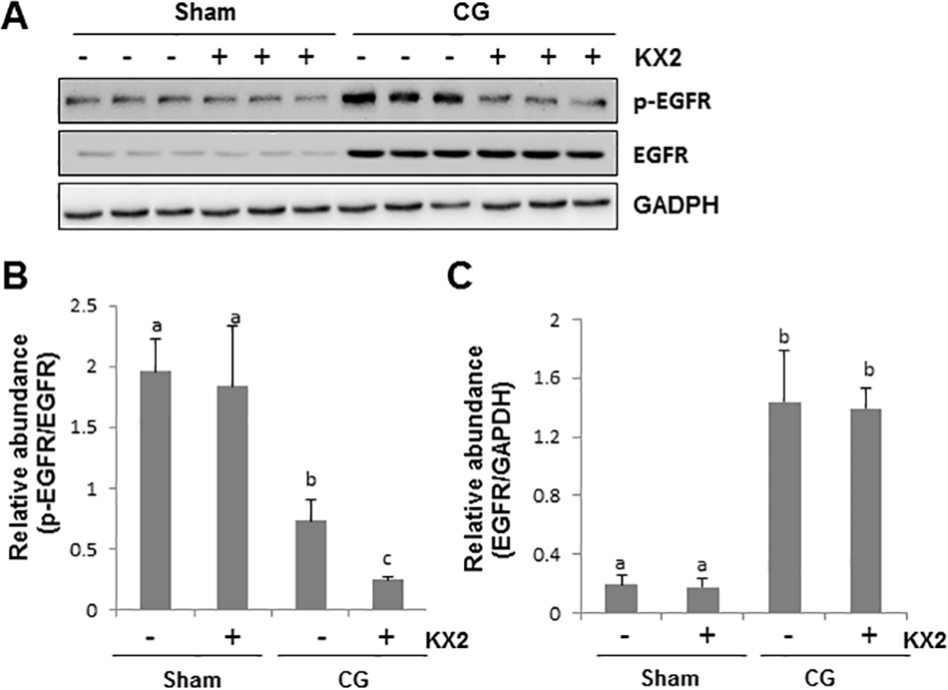
KX2-391 inhibits EGFR phosphorylation in the injured peritoneum Peritoneal membrane was collected at 21 days after CG injury with or without administration of KX2-391(KX2) (5 mg/kg, daily) (**A**–**C**). (A) The peritoneal tissue lysates were subjected to immunoblot analysis with specific antibodies against p-EGFR, EGFR, or GAPDH. (B) Expression levels of p-EGFR were quantified by densitometry and normalized with EGFR. (C) Expression levels of EGFR were quantified by densitometry and normalized with GAPDH. Data are means ± SEM (*n* = 6).Bars with different letters (a–c) are significantly different from one another (*P* < 0.05).

Our recent studies have shown that EGFR activation is critically involved in the development and progression of peritoneal fibrosis [8]. Since Src can mediate EGFR transactivation in response to some active substances such as Ang II [27, 28], we examined the effect of KX2-391 on the phosphorylation of EGFR in the peritoneum after CG injury. As shown in Figure 6, the basal level of phospho-EGFR and total EGFR was detected in the peritoneum of sham-operated animals and CG injury increased phosphorylation of EGFR. KX2-391 administration markedly reduced its phosphorylation to the basal level. Although expression levels of total EGFR also increased in the peritoneum after CG injury, KX2-391 treatment did not affect its expression. These data suggest that Src plays an essential role in CG injury-induced activation, but not in the expression of EGFR.

### Src mediates activation of STAT3 and Akt in the peritoneum after CG injury

**Figure 7:**
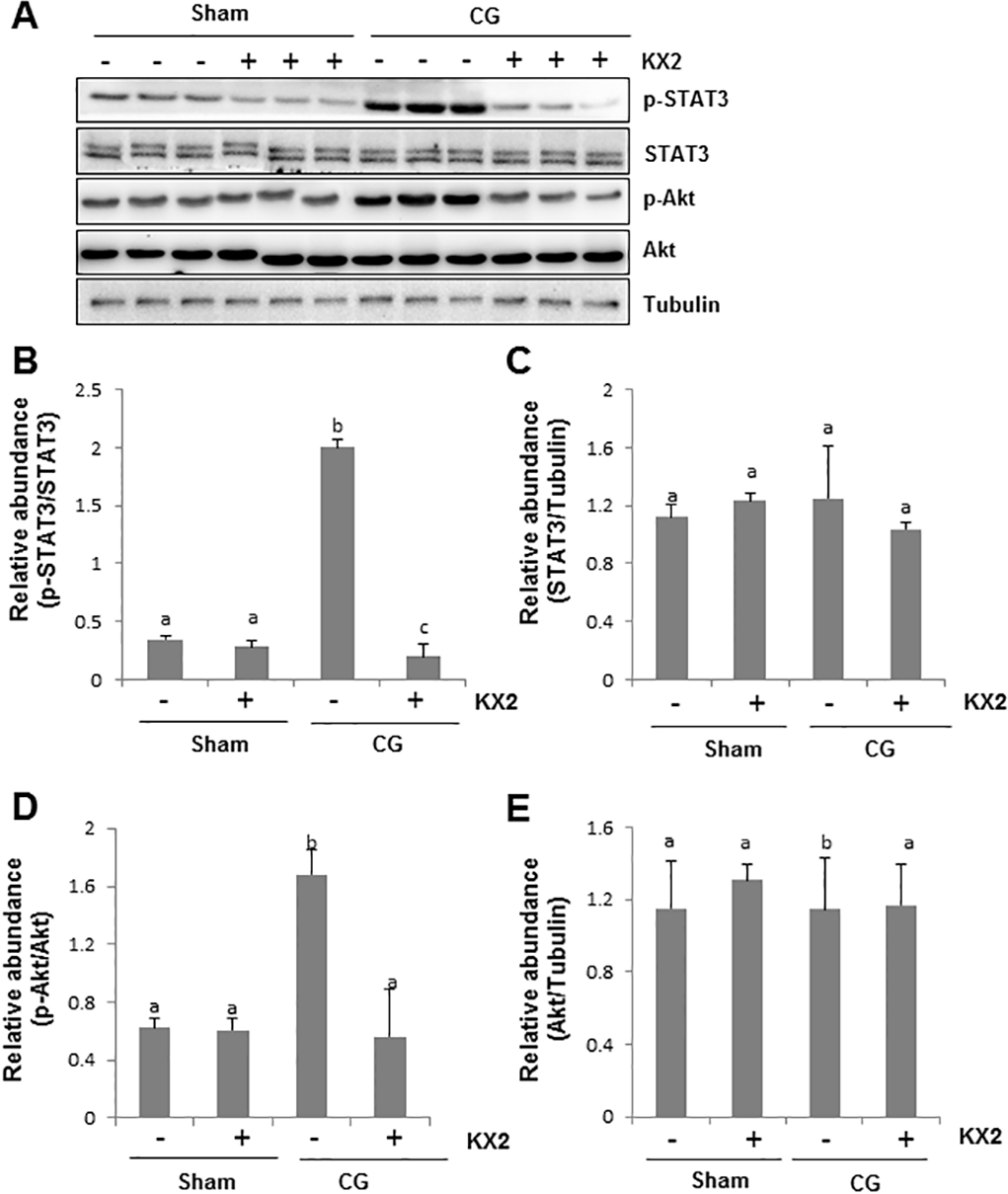
KX2-391 inhibits STAT3 and AKT phosphorylation in the injured peritoneum Peritoneal membrane was collected at 21 days after CG injury with or without administration of KX2-391(KX2) (**A**–**E**). (A) Peritoneum tissue lysates were subjected to immunoblot analysis with specific antibodies against p-STAT3, STAT3, p-AKT, AKT, or Tubulin. (B) Expression levels of p-STAT3 were quantified by densitometry and normalized with STAT3. (C) Expression levels of STAT3 were quantified by densitometry and normalized with Tubulin. (D) Expression level of p-AKT was quantified by densitometry and normalized with AKT. (E) Expression levels of AKT were quantified by densitometry and normalized with Tubulin. Data are means ± S.E.M. (*n* = 6). Bars with different letters (a–c) are significantly different from one another (*P* < 0.05).

It has been reported that STAT3 plays an important role in mediating the activation of renal interstitial fibrosis in the kidney after ureteral obstruction [29–31]. PI3K/Akt signaling also modulates TGF-β1-induced EMT in renal tubular cells and the peritoneum [32, 33]. To determine whether Src-mediated peritoneal fibrosis is related to these two signaling pathways, we also examined the effect of Src inhibition on the phosphorylation and expression of STAT3 and Akt. As shown in Figure 7A, 7B, 7D, the phosphorylated levels of STAT3 and Akt were increased in CG injured peritoneum, and KX2-391 administration reduced their phosphorylation. CG injury and KX2-391 did not affect expression of total STAT3 and Akt (Figure 7C and 7E). Thus, these data illustrate that activation of STAT3 and Akt signaling pathways are subject to Src regulation in the peritoneum undergoing fibrosis and suggest that these two pathways may translate Src activation to peritoneal fibrosis.

### Src inhibition abrogates NF-κB phosphorylation in the peritoneum after CG injury

**Figure 8:**
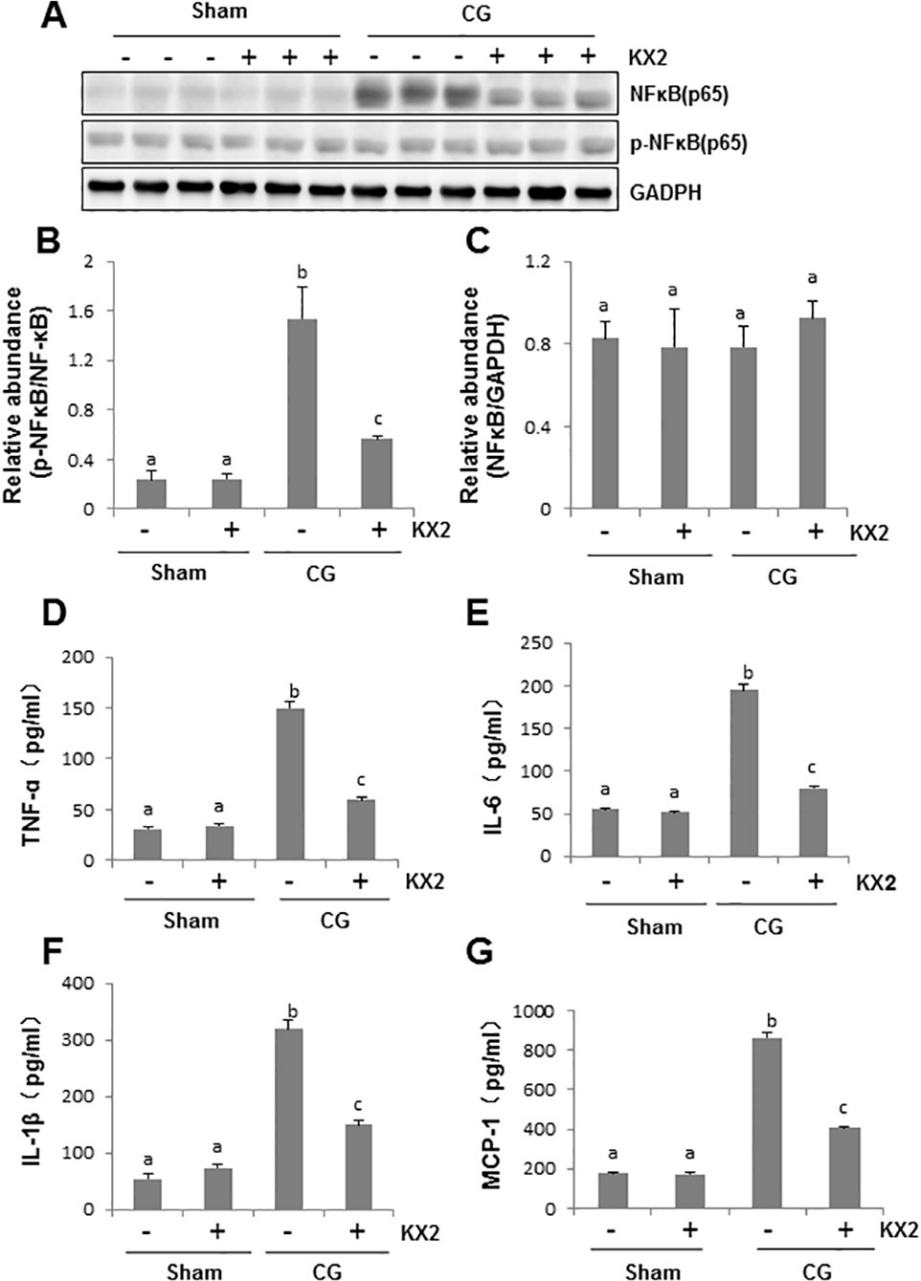
KX2-391 suppresses NF-κB(p65) phosphorylation and production of multiple proinflammatory cytokines/chemokines in the injured peritoneum Peritoneal membrane was collected at 21 days after CG injury with or without administration of KX2-391(KX2) (**A**–**G**). (A) Peritoneum tissue lysates were subjected to immunoblot analysis with specific antibodies against p-NF-κB (p65), NF-κB(p65) or NADPH. (B) Expression level of p-NFκB(p65) was quantified by densitometry and normalized with NFκB(p65). (C) Expression levels of NFκB(p65)were quantified by densitometry and normalized with GAPDH. Graphs show the expression level of TNF-α (D), IL-6 (E), IL-1β (F), and MCP-1 (G) by ELISA. Data are means ± S.E.M. (*n* = 6). Bars with different letters (a–c) are significantly different from one another (*P* < 0.05).

NF-κB signaling pathway plays an important role in regulating angiogenesis and inflammation in peritoneal fibrosis [34]. Thus, we examined the effect of Src inhibition on the phosphorylation of NF-κB. As shown in Figure 8A–8C, phosphorylated NF-κBp60 was barely detectable in the sham-operated and KX2-391-treated rat peritoneum. Injection of CG increased expression of the NF-κBp60 phosphorylated form, but not its total levels. Treatment with KX2-391 significantly inhibited NF-κB phosphorylation in the CG injured peritoneum. This data indicates that Src functions as a regulator of NF-κB signaling pathways during peritoneal fibrosis, and suggests that NF-κB may mediate Src-induced proinflammatory responses and angiogenesis.

### Src inhibition suppresses production of multiple inflammatory cytokines in the peritoneum after CG injury

Increased expression of proinflammatory cytokines/chemokines in the submesothelial compact zone is regarded as typical pathological changes in the fibrotic peritoneum [35]. To demonstrate whether Src mediates these responses after CG injury, we examined the expression of TNF-ɑ, IL-6, IL1-β, and MCP-1 by ELISA assay. The expression of all these cytokines/chemokines in the peritoneum was significantly elevated after CG injury and inhibited by KX2-391 treatment (Figure 8D–8G). Therefore, Src may also be an important regulator of proinflammatory responses during peritoneal fibrosis.

### Src inhibition suppresses infiltration of macrophages in the peritoneum after CG injury

**Figure 9:**
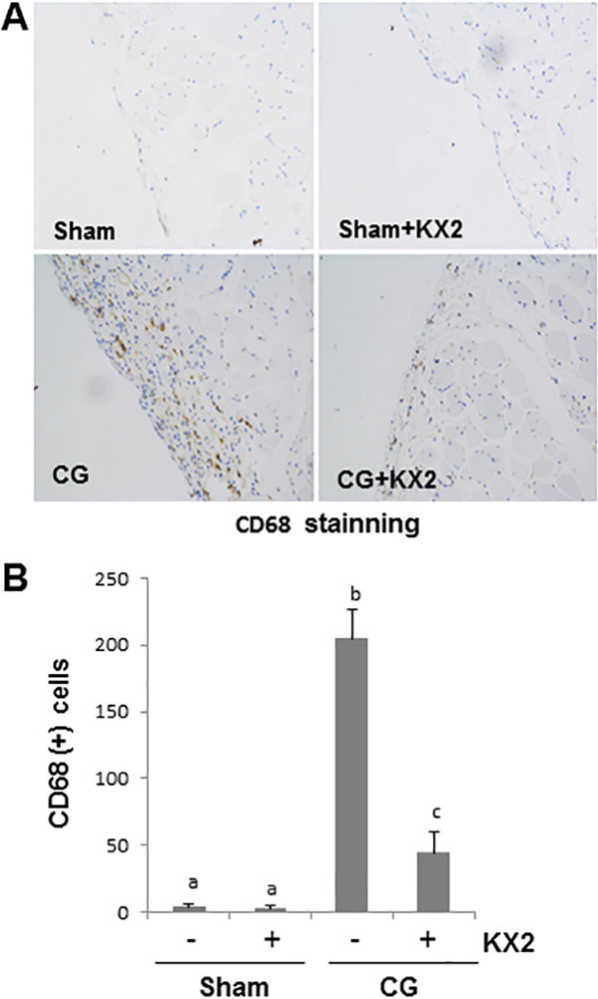
KX2-391 inhibits peritoneal macrophage infiltration in the injured peritoneum Peritoneal membrane was collected at 21 days after CG injury with or without administration of KX2-391(KX2) (**A**, **B**). (A) Photomicrographs illustrate immunohistochemical staining of CD68 in the submesothelial compact zone. (B) The number of CD68-positive cells (yellow) was calculated from 10 random fields (200 X) of six rat peritoneal samples. Data are means ± S.E.M. (*n* = 6). Bars with different letters (a–c) are significantly different from one another (*P* < 0.05).

One of the key pathologic changes in peritoneal fibrosis is the infiltration of macrophages in the thickened submesothelial compact zone [35]. To elucidate the effect of KX2-391 on macrophage infiltration, we conducted immunohistochemistry staining using an antibody against CD68, a marker of active macrophages. We found that macrophage infiltration increased in the submesothelial layer of rat peritoneum after CG injury, but was significantly reduced following KX2-391 treatment (Figure 9A, 9B). Therefore, Src activation is required for the accumulation of macrophages in the fibrotic peritoneum.

### Src inhibition attenuates angiogenesis and expression of VEGF in the peritoneum after CG injury

**Figure 10:**
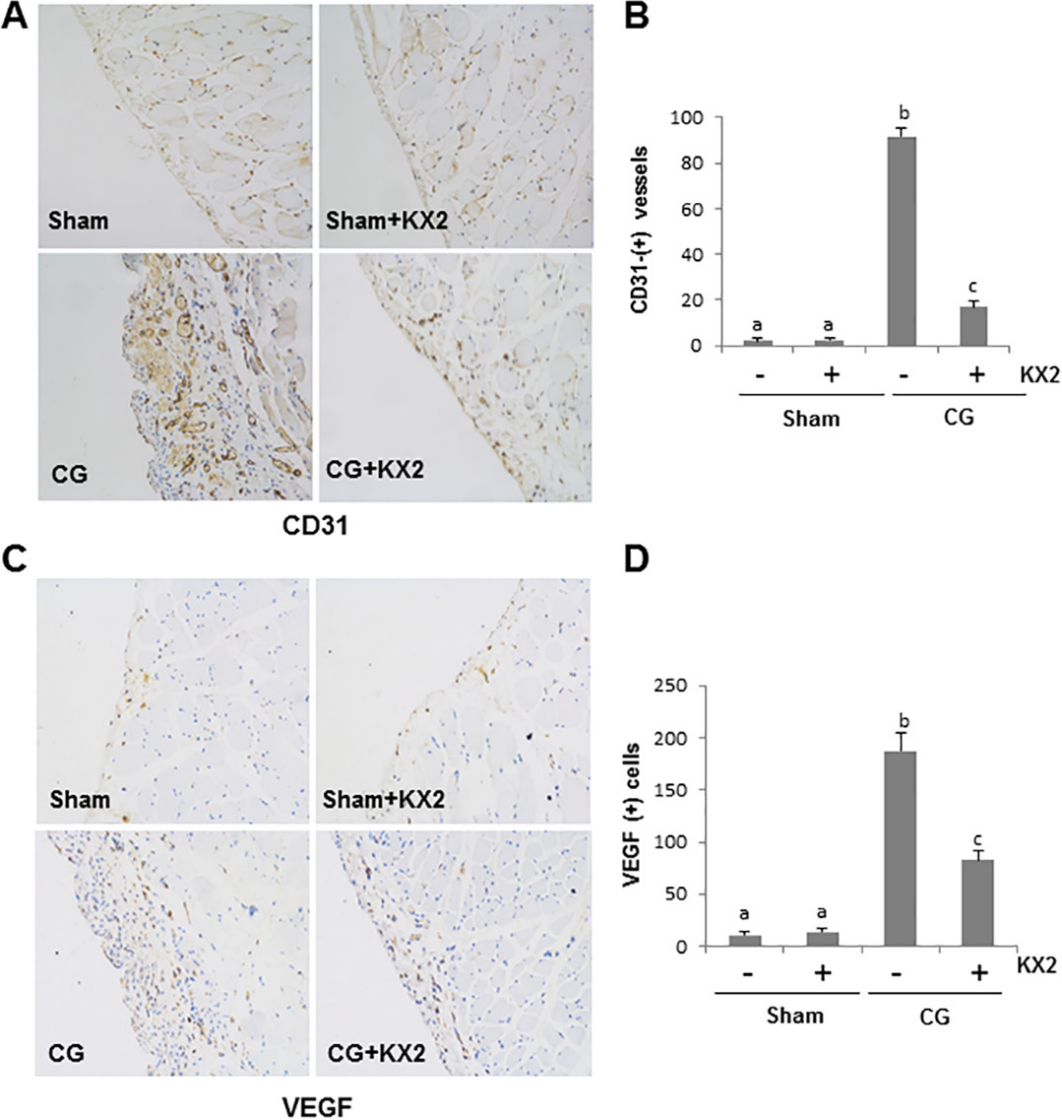
KX2-391 inhibits CD31 and VEGF expression in the injured peritoneum Peritoneal membrane was collected at 21 days after CG injury with or without administration of KX2-391(KX2) (**A**–**D**). Photomicrographs illustrate immunohistochemical staining of CD31 and VEGF (yellow) in the submesothelial compact zone (A, C). The number of CD31-positive vessels (B), or VEGF-positive cells (D) was accounted from 10 random fields (X200) of six rat peritoneal samples. Data are means ± S.E.M. (*n* = 6). Bars with different letters (a-c) are significantly different from one another (*P* < 0.05).

Long-term PD is associated with angiogenesis [36], a pathological event associated with overproduction of VEGF [37]. To determine the role of Src in this process, we examined the expression of endothelial cell marker CD31 and VEGF in the peritoneum. The number of CD31(+) vessels in the peritoneum markedly increased after CG injection compared with the sham group. Administration of KX-391 significantly suppressed this response (Figure 10A and 10B). An increase in VEGF-positive cells was also observed in the peritoneal membrane of rats injected with CG, and KX2-391 treatment largely reduced this population of cells (Figure 10C and 10D). These data indicate that blocking Src with KX2-391 can inhibit neo-angiogenesis, perhaps through a mechanism involved in the suppression of VEGF expression.

### Src activity is required for serum-induced EMT in cultured human peritoneal mesothelial cells

**Figure 11:**
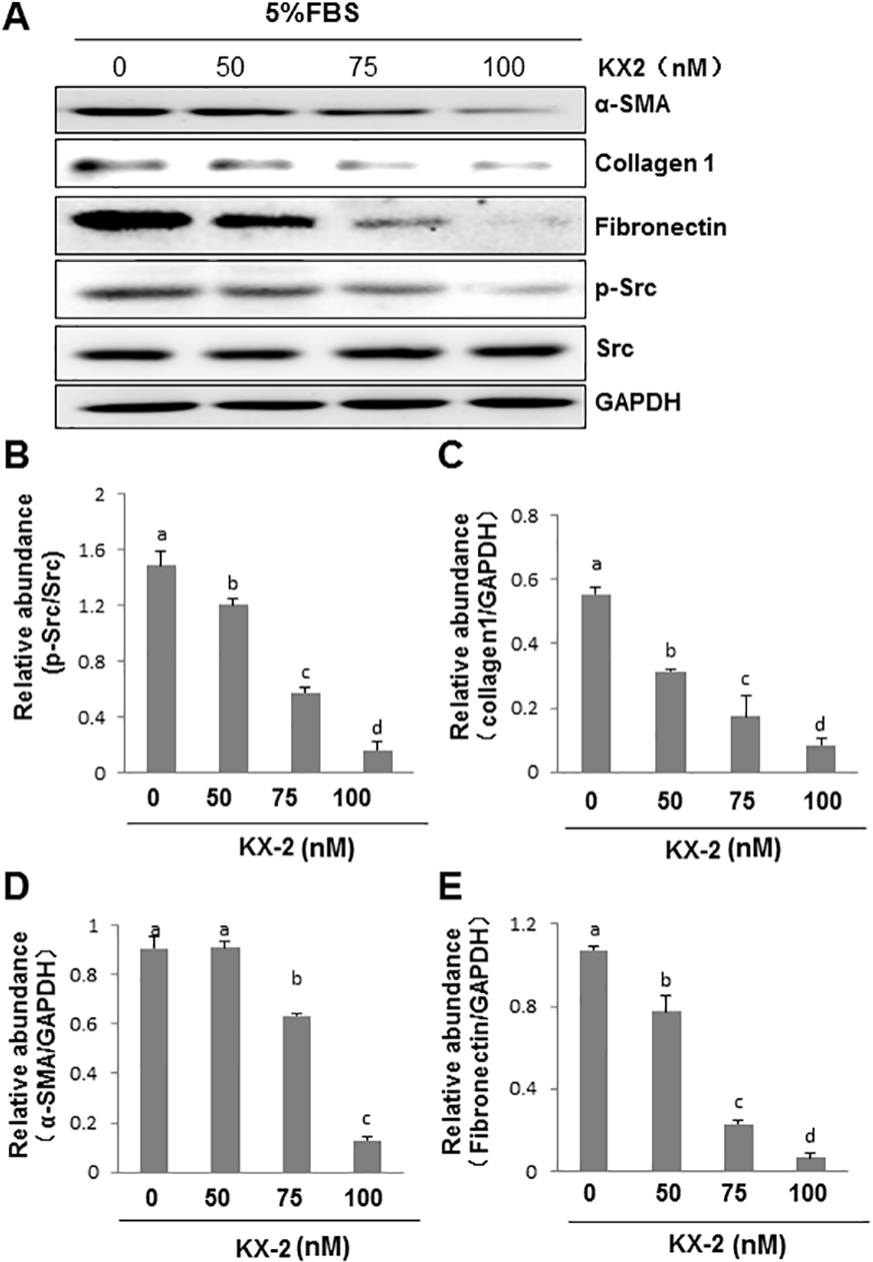
KX2-391 inhibits expression of fibronectin, collagen 1 and α-SMA in cultured human peritoneal mesothelial cells HPMCs cultured in DMEM-F12 containing 5% fetal bovine serum (FBS) were exposed to KX2-391(KX2) (50, 75, and 100 nM) for 24 h. (**A**) Cell lysates were subject to immunoblot analysis with antibodies to collagen 1, **α-SMA**, fibronectin, phospho-Src, Src, and GAPDH. (**B**) Expression levels of p-Src were quantified by densitometry and normalized with Src. (**C**–**E**) Expression levels of indicated proteins were quantified by densitometry and normalized with GAPDH. Data are means ± S.E.M. Bars with different letters (a–d) are significantly different from one another (*P* < 0.05).

Peritoneal mesothelial cells have been reported to undergo epithelial-mesenchymal transition (EMT) in response to injury, a cellular event associated with peritoneal fibrosis [38]. To understand the role of Src in the EMT of peritoneal mesothelial cells, normally cultured human peritoneal mesothelial cells (HPMCs) were exposed to different doses of KX2-391 and expression of, α-SMA, fibronectin and collagen 1 were examined. As shown in Figure 11, α-SMA, fibronectin and collagen 1, three hallmarks of EMT, were expressed in cultured HPMCs with serum. KX2-391 at 50 nM exhibited an inhibitory effect on the expression of collagen 1 and fibronectin. At 100 nM, KX2-391 largely blocked the expression of all of these three proteins. Phosphorylated Src was clearly detected in cultured HPMCs. Treatment with KX2-391 dose-dependently inhibited the Src phosphorylation with a complete inhibition at 100 nM. Collectively, these data illustrate that Src is a key signaling molecule that mediates the EMT in HPMCs.

### Silencing Src with siRNA inhibits EMT in cultured human peritoneal mesothelial cells

**Figure 12:**
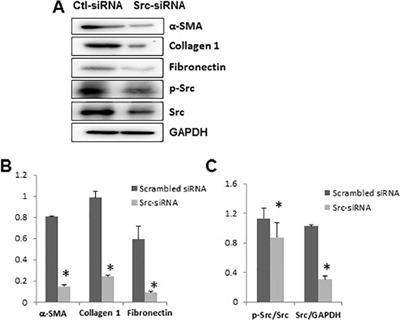
Silencing of Src with small interfering RNA (siRNA) inhibits expression of fibronectin, collagen 1 and α-SMA in cultured human peritoneal mesothelial cells HPMCs were transfected with scrambled siRNA or siRNA specific to Src and incubated for 48 h in DMEM-F12 with 5% fetal bovine serum. (**A**) Cells were harvested and cell lysates were subject to immunoblot analysis with antibodies to collagen 1, α-SMA, fibronectin, phospho-Src, Src, and GAPDH. (**B**) Expression levels of indicated proteins were quantified by densitometry and normalized with GAPDH. (**C**) Expression levels of p-Src and Src were quantified by densitometry and then normalized with Src and GAPDH, respectively. Data are means ± S.E.M. Bars with*are significantly different from controls (*P* < 0.05).

To confirm the role of Src in regulating the EMT of peritoneal mesothelial cells, we further examined the effect of Src silencing on this process by transfection of small interfering RNA (siRNA) specifically targeting Src in HPMCs. As shown in Figure 12A and 12B, knockdown of Src blocked expression of α-SMA, fibronectin, and collagen 1 in HPMCs. Expression of total Src and its phosphorylated form (Tyr416) was remarkably decreased in HPMCs transfected with siRNA (Figure 12A and 12C). This further demonstrates the importance of Src in mediating the EMT of peritoneal mesothelial cells.

### Src mediates TGF-β1 induced EMT and production of collagen 1 *in vitro*

Since TGF-β is a potent cytokine that stimulates EMT and induces peritoneal fibrosis [39], we further examined whether Src would also play a role in TGF-β stimulated EMT in HPMCs *in vitro*. Exposure of serum starved HPMCs to TGF-β1 (2 ng/ml) for 24 h resulted in increased expression of α-SMA, fibronectin, and collagen 1. KX2-391 treatment also reduced expression of all of them with maximum inhibition at 100 nM (Supplementary Figure 1). Similarly, KX2-391 inhibited TGF-β1-induced Src phosphorylation, whereas total Src expression was not affected by this agent. Thus, it appears that Src is also coupled to the TGF-β signaling pathway to regulate the EMT of peritoneal mesothelial cells.

## DISCUSSION

The anti-fibrotic effects of Src inhibition have been reported in several experimental models of fibrotic diseases such as idiopathic pulmonary fibrosis [19, 40], liver fibrosis [20], renal fibrosis [21–23], and systemic sclerosis [18]. However, it remains unknown whether inhibition of Src also ameliorates peritoneal fibrosis. In this study, we demonstrated that (i) Src kinase is activated in cultured HPMCs and in the fibrotic peritoneum; (ii) pharmacological inhibition with KX2-391 or silencing of Src reduces the EMT of HPMCs; (iii) administration of KX2-391 reduces peritoneal fibroblast activation and attenuates accumulation of ECM in the rat model of peritoneal fibrosis induced by CG injury. To our knowledge, this is the first study to demonstrate the beneficial effects of Src inhibition on peritoneal fibrosis and reveal Src kinase as a key mediator in peritoneal fibrosis.

The CG model is an ideal model for examining the efficacy of potential therapeutic reagents for treating peritoneal fibrosis [41]. In this model, we observed that CG injury induces a time-dependent increase in the phosphorylation of Src at Tyr 416 (activation) in the peritoneum, which parallels the time course of ECM accumulation and fibroblast activation. Immunostaining indicated that the active Src form was most localized in α-SMA (+) cells in the injured peritoneum, consistent with our demonstration that Src activity is required for the activation of peritoneal myofibroblasts and fibrosis. In support of this statement, our *in vitro* studies also demonstrated that blocking or silencing Src resulted in inhibition of EMT as evidenced by decreased expression of α-SMA as well as collagen 1 and fibronectin.

Currently, the mechanism of Src mediated peritoneal fibrosis is not fully understood. As the TGF-β/Smad3 signaling pathway has been reported to be implicated in the pathogenesis of peritoneal fibrosis [37, 38], we examined the effect of Src inhibition on the activation of this pathway. Our results show that the CG-injured fibrotic peritoneum is accompanied by increased expression of TGF-β receptor I and II as well as phosphorylation of Smad3, a key mediator of TGF-β signaling. Administration of KX2-391 dramatically inhibited these responses. In addition, either blockage of Src with KX2-391 or silencing of it with siRNA reduced TGF-β1 induced expression of α-SMA, fibronectin, and collagen 1 in HPMCs. This suggests that Src is coupled to the TGF-β/Smad signaling to induce the EMT of HPMCs and promote peritoneal fibrosis. Currently, it remains unclear how Src induces activation of the TGF-β1/Smad3 signaling pathway. One possibility is that Src may regulate this pathway through activation of AKT since Src can directly induce AKT phosphorylation and AKT can induce phosphorylation of ubiquitin specific protease 4 (USP4), a deubiquitinating enzyme that prevents TGF-β receptor I degradation [42]. Another possibility is that Src may activate the TGF-β1/Smad3 pathway through TGF-β production. In this regard, it has been reported that Src mediates Angiotensin II induced production of TGF-β1 via transactivation of EGFR in the fibrotic kidney [17]. See comment in PubMed Commons below

EGFR is a tyrosine kinase that can be activated with or without the requirement for ligands. Src is one of the key signaling molecules that can directly induce EGFR phosphorylation at Tyr-845 [43]. On the other hand, Src can also indirectly activate EGFR through promoting release of soluble forms of EGFR ligands via a mechanism involved in the activation of matrix metalloproteinases [44]. Since our recent studies have demonstrated that EGFR activation is critically involved in the development of peritoneal fibrosis [8], there is the possibility that Src may induce peritoneal fibrosis through activation of EGFR. To test this hypothesis, we examined the effect of KX2-391 on EGFR phosphorylation in the peritoneum after CG injury and found that treatment with KX2-391 reduced EGFR phosphorylation to the basal level. These data, together with our recent observations that blocking EGFR inhibited peritoneal fibrosis, suggests that EGFR may play an essential role in transducing Src activation to peritoneal fibrosis.

Src induces activation of multiple intracellular signaling pathways including the phosphatidyl inositol 3 kinase (PI3K)/Akt pathway and STAT3 pathways [13]. The involvement of these two signaling pathways in tissue fibrosis has been extensively studied [29, 45]. PI3K/Akt can modulate TGF-β1 induced EMT in renal tubular cells [32]. Treatment with LY294002 (an inhibitor of PI3K/Akt) significantly inhibits EMT transformation in renal tubular epithelial cells [46, 47]. Recent studies also demonstrated that the PI3K/Akt pathway mediates EMT in a model of PD and HPMCs [33]. Similarly, STAT3 is an important transcriptional regulator associated with the expression of ECM and/or cytokines/chemokines. Our recent studies demonstrated that STAT3 is involved in the renal fibroblast activation and renal fibrogenesis [31]. Blocking JAK2, the direct upstream activator of STAT3, also attenuated peritoneal membrane inflammation, fibrosis, and hypervascularity [48]. In line with these results, our study showed that the CG-injured fibrotic peritoneum is accompanied by phosphorylation of STAT3 and Akt. Treatment with KX2-391 inhibits phosphorylation of STAT3 and Akt. Thus, in addition to the TGF-β/Smad3 signaling pathway, Src may also regulate the EMT and peritoneal fibrosis through activation of the PI3K/Akt and STAT3 pathways.

Inflammation is one of the main pathological processes contributing to peritoneal fibrosis during long-term PD [49, 50]. The character of inflammatory response includes expression of multiple cytokines/chemokines and infiltration of macrophages. In this study, we found that the expression of four pro-inflammatory factors, including TNF-α, MCP-1, IL-β1 and IL-6, increased in the peritoneum injured by CG. Blocking Src inhibited expression of all of them and reduced infiltration of macrophages to the peritoneum after CG injury. Given that NF-κBp60 is a transcriptional factor that promotes expression of multiple pro-inflammatory factors, and KX2-391 treatment suppressed NF-κBp60 phosphorylation, the anti-inflammatory response may also be an important mechanism by which Src inhibition offers peritoneal protection.

Angiogenesis is another important promoter in the progression of peritoneal fibrosis and ultrafiltration failure [12, 51]. Peritoneal expression of VEGF is correlated with the degree of angiogenesis, and inhibition of VEGF can inhibit peritoneal angiogenesis in the rat mode of peritoneal fibrosis [52–54]. In the present study, we examined the effect of KX2-391 on the expression of VEGF and found that KX2-391 was effective in suppressing its expression in the peritoneum of CG injured rats. In addition, we observed that KX2-391 treatment inhibited expression of CD31, a marker of vascular endothelial cells. Therefore, Src inhibitors may also exhibit their anti-fibrotic effects in the peritoneum through inhibition of angiogenesis.

Given that Src is activated by multiple profibrotic growth factors/cytokines and is involved in many pathologucal conditions, chemical inhibitors targeting Src kinases have been developed for the treatment of many diseases, in particular, tumors. Among Src inhibitors, dasatinib has been approved by the U.S. Food and Drug Administration to treat chronic myeloid leukemia [55]. Other SFK inhibitors such as saracatinib (AZD0530) and bosutinib (SKI-606) are also under clinical trials for the treatment of solid tumors ( www.clinicaltrials.gov). KX2–391 is a novel Src inhibitor that targets the peptide substrate–binding site rather than the ATP-binding site mechanism that is used by other Src inhibitors [56]. A phase I study of KX2–391 demonstrated biologic activity in pancreatic and prostate cancer patients [26]. Currently, there is no available therapy for halting or preventing peritoneal fibrosis, it would thus be beneficial to initiate clinical trials to investigate the therapeutic effect of KX2-391 in patients with peritoneal fibrosis during PD in the future.

**Figure 13:**
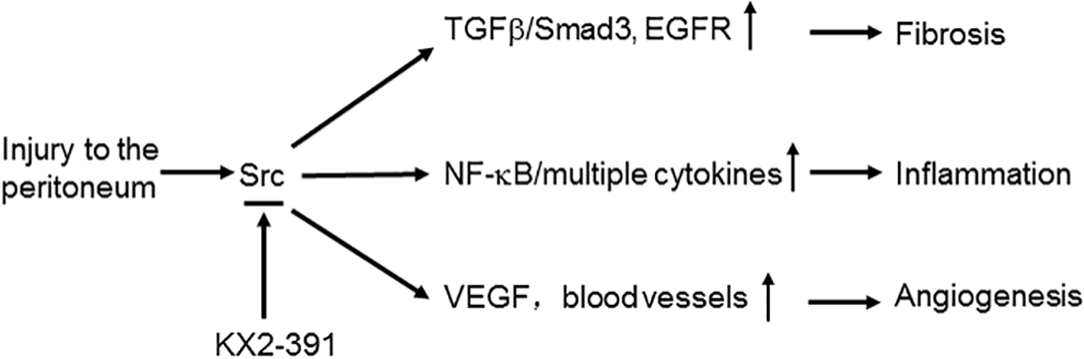
Mechanisms of Src mediated peritoneal fibrosis Injury to the peritonium induces activation of Src, which subsequently leads to activation of multiple profibrotic signaling pathways (i.e. TGFβ/smad3, EGFR), induction of proinflammatory responses (i.e. activation of NF-kB, production of multiple cytokines) and promotion of angiogenesis via increasing VEGF production. All these responses can be inhibited by KX2-391, a highly selective Src inhibitor.

In summary, Src is activated in CG-induced peritoneal fibrosis and blocking Src can prevent progression of peritoneal fibrosis. These beneficial effects may be exerted through the inhibition of multiple profibrotic signaling pathways, inflammatory responses, and angiogenesis (Figure 13). Thus, targeting Src may represent a novel approach to prevent the development of peritoneal fibrosis in PD patients.

## MATERIALS AND METHODS

### Reagents and antibodies

Antibodies to p-NF-κB (p65), NF-κB (p65), p-STAT3, STAT3, p-Smad3, Smad3, p-Akt, Akt, p-Src, Src and p-EGFR were purchased from Cell Signaling Technology (Danvers, MA). Antibodies to fibronectin, collagen 1(A2), GAPDH (glyceraldehyde 3-phosphate dehydrogenase), EGFR, CD31 and VEGF were purchased from Santa Cruz Biotechnology, Inc. (Santa Cruz, CA). Antibody to CD68 was purchased from Boster (Wuhan, China). Antibody toα-Tubulin was purchased from TransGen Biotech (Beijing, China). TNF-α, IL-1β, MCP-1, IL-6, TGF-β1enzyme-linked immunosorbent assay (ELISA) kits were from R&D systems (Minneapolis, MN). KX2-391 was purchased from Selleck Chemicals LLC (Houston, TX, USA). α-SMA, Chlorhexidin gluconate (CG) and all other chemicals were from Sigma-Aldrich (St. Louis, MO).

### Establishment of rat peritoneal fibrosis models and KX2-391 administration

The peritoneal fibrosis model was established in male Sprague-Dawley rats that weighed 200 ± 10 g (Shanghai Super -- B&K laboratory animal Corp. Ltd) as described in our previous study [57]. Briefly, peritoneal fibrosis in rats was generated by daily intraperitoneal injection of 0.1% gluconate chlorhexidine. Control rats were injected with an equal volume of 0.9% saline. To explore the effect of Src inhibition on peritoneal fibrosis, KX2-391 was oral administrated at a dose of 5 mg/kg in 50 μl DMSO at the first day of CG injection and then given daily. Animals were divided into four groups (*n* = 6): the sham group with either DMSO or KX2-391; the peritoneal fibrosis group induced by 0.1% CG with either DMSO or KX2-391. At 21 days, rats were killed and the parietal peritoneum was harvested for further analysis. All the experiments were conducted in accordance with the animal experimentation guideline of Tongji University School of Medicine, China.

### Cell culture and treatments

Human peritoneal mesothelium cells (HPMCs) were cultured in Dulbecco's modified eagle's medium (DMEM) (Sigma-Aldrich, St. Louis, MO) containing 5% fetal bovine serum (FBS), 1% penicillin and streptomycin in an atmosphere of 5% CO_2_ and 95% air at 37°C. To determine the effects of KX2-391 on HPMCs, KX2-391 was directly added to subconfluent HPMCs and then incubated for the indicated time as indicated in Figure legends. For TGF-β1 treatment, cells were starved for 24 h by incubation with 0.5% FBS containing DEME and then exposed to TGF-β1 (10 ng/ml) for 24 h in the absence or presence of KX2-391 (100 nM) before being harvested for protein analysis.

### Transfection of siRNA into cells

Small interfering RNA (siRNA) specific for Src was obtained from Santa Cruz Biotechnology, Inc. (Santa Cruz, CA). HPMCs were seeded to 50–60% confluence in antibiotic-free medium and grown for 24 h. Then cells were transfected with 100 nM siRNA using the Lipofectamine RNAiMAX reagent (Invitrogen, Carlsbad, CA). As a control, 100 nM scrambled control siRNA was also transfected to HPMCs in separate dishes. After transfection, cells were cultured for 6 hours in DMEM with 0.5% FBS for 6 hours and then changed to DMEM with 5% FBS and cultured for an additional 48 h in before cell lysates were prepared for immunoblot analysis.

### Immunoblot analysis

Immunoblot analysis of peritoneum tissue samples was conducted as described previously [57]. The densitometry analysis of immunoblot results was conducted by using NIH Image software (National Institutes of Health, Bethesda, MD).

### Morphological studies of peritoneum

Formalin-fixed peritoneum were embedded in paraffin and prepared in 3-μm-thick sections. For evaluation of peritoneal fibrosis, Masson trichrome staining was performed according to the protocol provided by the manufacturer (Sigma, St. Louis, MO). The collagen tissue area (blue color) was quantitatively measured using Image Pro-Plus software (Media-Cybernetics, Silver Spring, MD, USA) by drawing a line around the perimeter of the positive staining area. The average ratio to each microscopic field (200×) was calculated and graphed. The thickness of the sub-mesothelial tissue was evaluated (in micrometers), and the average of ten independent measurements was calculated for each section (×200 magnification).

### Immunohistochemical Staining

Immunohistochemical staining was conducted based on the procedure described in our previous studies [57]. For quantitative assessment, the positive staining area was measured by Image Pro-Plus Software, and the average ratio to each microscopic field (200×) was calculated and graphed. Immunofluorescent staining was carried out according to the procedure described in our previous studies [19]. Images were taken using a Zeiss 710 Duo microscope.

### ELISA detection

ELISA detection of TGF-β1, MCP-1, IL-1β, TNF-α, and IL-6 protein was performed in accordance with the manufacturer's instructions.

### Statistical analysis

Samples from six animals in each group were used for all the experiments. Immunoblots and tissue histology images are representative of at least three experiments from six animals. Data depicted in graphs represent the means ± SEM for each group. For all the experiments, the differences between two groups were made using one-way analysis of variance (ANOVA) followed by the Tukey's test. Statistically significant difference between mean values was marked in each graph.

## SUPPLEMENTARY MATERIALS FIGURE


