# Supplementary material for: Targeting Src attenuates peritoneal fibrosis and inhibits the epithelial to mesenchymal transition
Source: Oncotarget. 2017 Aug 8;8(48):83872–89. doi: 10.18632/oncotarget.20040 (PMC5663561; doi:10.18632/oncotarget.20040)
Supplement: Supplementary file 1 [file oncotarget-08-83872-s001.pdf]

Targeting Src attenuates peritoneal fibrosis and inhibits the epithelial to mesenchymal transition

SUPPLEMENTARY MATERIALS

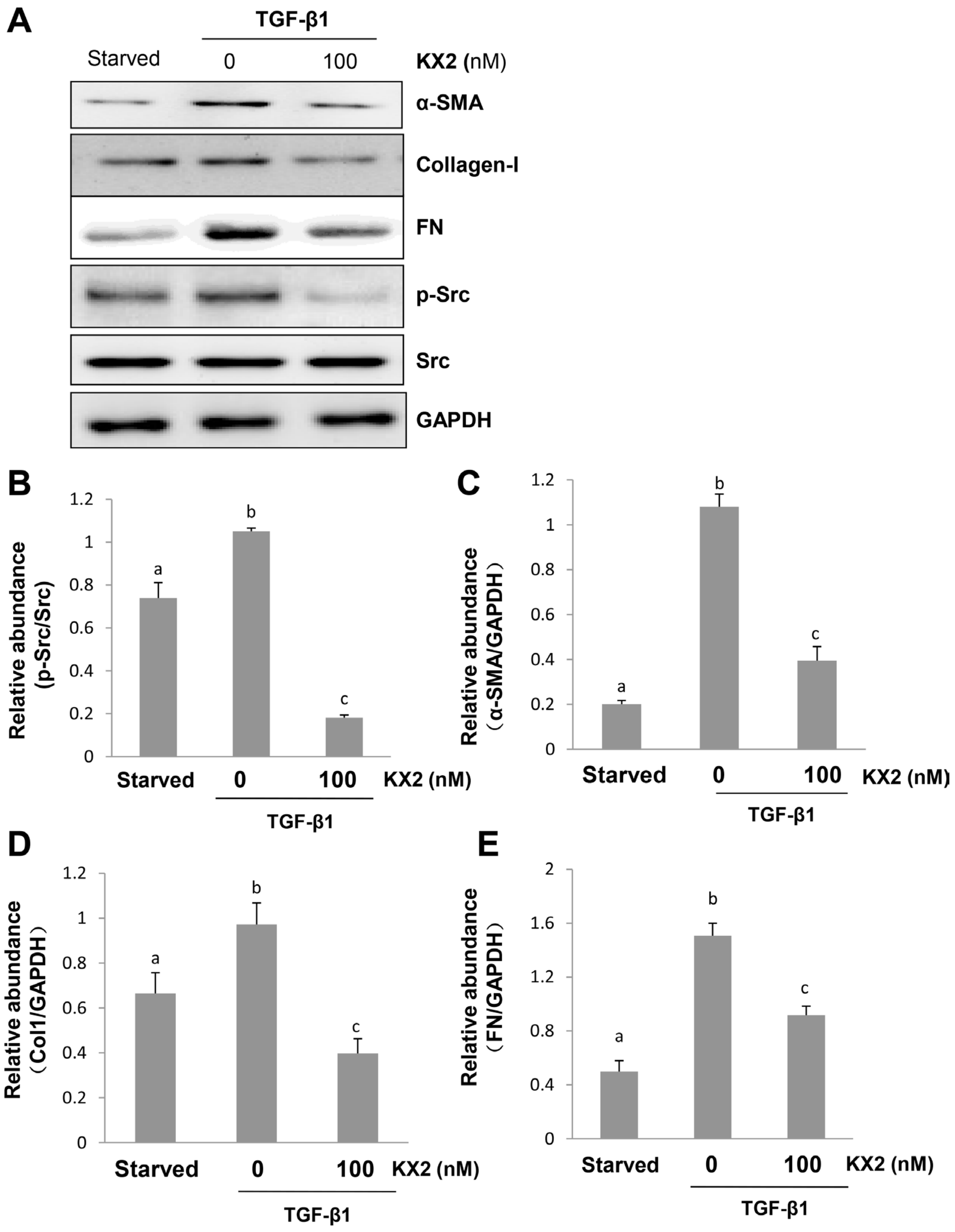

Supplementary Figure 1: KX2-391 reduces transforming growth factor-β1 (TGF-β1)-induced expression of fibronectin (FN), collagen-I and α-SMA in cultured human peritoneal mesothelial cells. Serum-starved HPMCs were cultured with 2 ng/ml TGF-β1 for 24 h in the presence of KX2-391 (100 nM). (**A**) Cell lysates were subject to immunoblot analysis with antibodies to collagen 1, α-SMA, fibronectin, phospho-Src (p-Src), Src, and GAPDH. (**B**) Expression levels of p-Src were quantified by densitometry and normalized with Src. (**C–E**) Expression levels of indicated proteins were quantified by densitometry and normalized with GAPDH. Data are means ± S.E.M. Bars with different letters (a–c) are significantly different from one another ($P < 0.05$).
